# Altered Statistical Learning and Decision-Making in Methamphetamine Dependence: Evidence from a Two-Armed Bandit Task

**DOI:** 10.3389/fpsyg.2015.01910

**Published:** 2015-12-18

**Authors:** Katia M. Harlé, Shunan Zhang, Max Schiff, Scott Mackey, Martin P. Paulus, Angela J. Yu

**Affiliations:** ^1^Department of Psychiatry, University of California San DiegoLa Jolla, CA, USA; ^2^Department of Cognitive Science, University of California San DiegoLa Jolla, CA, USA; ^3^Department of Psychiatry, Vanderbilt UniversityNashville, TN, USA; ^4^Department of Psychiatry, University of VermontBurlington, VT, USA; ^5^Laureate Institute for Brain ResearchTulsa, OK, USA

**Keywords:** Bayesian model, decision-making, reward processing, methamphetamine stimulant, addiction, multi-armed bandit task

## Abstract

Understanding how humans weigh long-term and short-term goals is important for both basic cognitive science and clinical neuroscience, as substance users need to balance the appeal of an immediate high vs. the long-term goal of sobriety. We use a computational model to identify learning and decision-making abnormalities in methamphetamine-dependent individuals (MDI, *n* = 16) vs. healthy control subjects (HCS, *n* = 16), in a two-armed bandit task. In this task, subjects repeatedly choose between two arms with fixed but unknown reward rates. Each choice not only yields potential immediate reward but also information useful for long-term reward accumulation, thus pitting exploration against exploitation. We formalize the task as comprising *a learning* component, the updating of estimated reward rates based on ongoing observations, and a decision-making component, the choice among options based on current beliefs and uncertainties about reward rates. We model the learning component as iterative Bayesian inference (the Dynamic Belief Model), and the decision component using five competing decision policies: Win-stay/Lose-shift (WSLS), ε-Greedy, τ-Switch, Softmax, Knowledge Gradient. HCS and MDI significantly differ in how they learn about reward rates and use them to make decisions. HCS learn from past observations but weigh recent data more, and their decision policy is best fit as Softmax. MDI are more likely to follow the simple learning-independent policy of WSLS, and among MDI best fit by Softmax, they have more pessimistic prior beliefs about reward rates and are less likely to choose the option estimated to be most rewarding. Neurally, MDI's tendency to avoid the most rewarding option is associated with a lower gray matter volume of the thalamic dorsal lateral nucleus. More broadly, our work illustrates the ability of our computational framework to help reveal subtle learning and decision-making abnormalities in substance use.

## Introduction

Negotiating the tension between exploration and exploitation is an important aspect of cognitive processing, as actions leading to immediate reward may well-conflict with actions that aid the attainment of long-term goals. Daily examples include partying vs. studying, snacking vs. dieting, and relaxing vs. exercising. Understanding how the brain solves this exploration vs. exploitation problem is not only important for basic cognitive science, but also clinical neuroscience, where substance abusers are often faced with the choice between immediate high vs. long-term sobriety. A classical behavioral paradigm used to study the tradeoff between exploration and exploitation task is the multi-armed bandit task (Daw et al., 2006; Behrens et al., 2007; Erev et al., 2008; Gonzalez and Dutt, 2011; Hills and Hertwig, 2012; Zhang et al., 2014), in which subjects must make repeated choices among options (bandit arms) that yield rewards with fixed but unknown probabilities. Selecting different options may either maximize the immediate likelihood of receiving a reward, or the gain in information useful for long-term reward accumulation, thus creating a tension between exploitation and exploration. Bandit problems are widely used in psychology (Steyvers et al., 2009; Zhang and Yu, 2013a), decision neuroscience (Behrens et al., 2007; Cohen et al., 2007), and artificial intelligence (Kaelbling et al., 1996), to study the exploration-exploitation tradeoff. In this work, we use a Bayesian modeling framework to investigate how methamphetamine-dependent individuals differ from healthy controls in performing a two-armed bandit task.

### Methamphetamine dependence and cognitive deficits

Methamphetamine dependence (MD) is a serious public health concern (Panenka et al., 2013) associated with a high likelihood of relapse (Brecht and Herbeck, 2014). By 2008, nearly 25 million people worldwide were estimated to have used amphetamine/methamphetamine within the past year (Buxton and Dove, 2008), with abuse being particularly prevalent among younger age groups (Leland and Paulus, 2005). Importantly, executive deficits, most prominent in cognitive control and decision-making paradigms, have been consistently observed in stimulant abusers and implicated in the progression of abuse to dependence (Paulus et al., 2005; Clark et al., 2012; Gowin et al., 2014). Identifying precise neurocognitive markers of such alterations may therefore not only improve our understanding of how neurochemical changes in MD affect decision-making, but it may help identify robust neural predictors of relapse and treatment response.

### Reward processing impairments in MDI

Although much attention has been given to understanding alterations in impulse control among addicted individuals, neuroimaging studies in both animals and humans point to equally important disturbances in incentive salience and valuation (Goldstein and Volkow, 2011). Specifically, while stimulant abusers exhibit enhanced sensitivity to drugs and drug cues, they show decreased responsiveness to other types of rewards, including secondary reinforcers such as money, which is associated with decreased activation in the orbitofrontal and ventromedial prefrontal cortex (Goldstein et al., 2007; Goldstein and Volkow, 2011). This decreased responsiveness to non-drug rewards is likely to underlie the anhedonic symptoms consistently observed in drug dependence (Koob and Le Moal, 2001), including stimulant dependence (Leventhal et al., 2010), and may promote difficulties regulating stress and negative affect in addicts (London et al., 2004; Tabibnia et al., 2011).

### Learning deficits in MDI

Stimulant dependent individuals also demonstrate impairments in learning new information and in using this knowledge to guide decisions. For instance, chronic amphetamine abusers are not as efficient at learning to avoid high penalty options in the Iowa Gambling Task (Rogers et al., 1999; van der Plas et al., 2009), a deficit shown to be proportional to years of abuse (Rogers et al., 1999). Consistent with this poor learning and difficulties “seeing the big picture,” MDI demonstrate a greater discounting of delayed rewards (Hoffman et al., 2006; Monterosso et al., 2007) and a more “myopic” strategy in prediction tasks, with stronger reliance on previous trial outcomes relative to the overall success rates of choice alternatives (Paulus et al., 2002, 2003). Interestingly, during risky decision-making, MDI also demonstrate hypo-activations in the dorsolateral prefrontal cortex (DLPFC) and anterior insula (Ersche et al., 2005; Paulus et al., 2005), brain regions playing an important role in supporting learning and retrieval of stimulus-response associations (Miller and Cohen, 2001; Bunge et al., 2005) and interoceptive function (Paulus and Stein, 2006), respectively.

Together, these findings suggest that MD is associated with impaired tracking and updating of action values and changing contingencies in the environment, which may promote more rigid decision-making strategies (Aron and Paulus, 2007). Combined with reward processing alterations, MDI might also be more likely to make suboptimal choices in complex multi-option environments. Disentangling and quantifying the respective impact of such deficits in these different components of choice behavior (e.g., learning vs. decision policy vs. reward salience) remains a challenge, given only coarse behavioral performance measures (such as monetary earnings or average choice probabilities). In contrast, a model-based approach with more sophisticated representation of individuals' internal computations and variables can perhaps uncover more subtle effects.

### A bayesian approach to understanding decision-making deficits in MDI

We have proposed that two separable computational components underlie human choice behavior in the bandit task (Zhang and Yu, 2013a,b): a learning component, the updating of internal knowledge and uncertainty based on successive observations (e.g., successes or failures to obtain reward from the chosen options); and a decision-making component, the selection of an action based on current beliefs and uncertainties about the reward availability at different options). Consistent with this framework, we hypothesize that alterations in one or both of these components could be observed in individuals with a substance disorder such as MDI.

Bayesian models provide a way to address the learning component by quantifying individuals' beliefs about their environment and the associated uncertainty. In this framework, decision-makers are assumed to continuously update their beliefs of the environment based on each new observation. Specifically, we can model statistical learning about reward rates using a version of the Dynamic Belief Model, or DBM (Yu and Cohen, 2009), which assumes subjects believe that environmental statistics can undergo discrete, unsignaled changes without warning. Although reward rates are actually fixed (but unknown) in the bandit task employed in this study, we surmise that individuals may still exhibit *sequential effects, a* persistent tendency to form expectations about upcoming stimuli based on recent trials, which we have shown to arise from the belief that environmental statistics are changeable rather than fixed (Yu and Cohen, 2009). We have shown that DBM accurately predicts sequential effects in a wide variety of behavioral tasks in which stimulus statistics are fixed: perceptual decision-making (Yu and Cohen, 2009), visual search (Yu and Huang, 2014), inhibitory control (Ide et al., 2013; Harlé et al., 2014), and multi-arm bandit tasks (Zhang and Yu, 2013a,b). To model the decision-making component, this Bayesian formulation can be naturally combined with various decision-making (i.e., action selection) models to infer individuals' learning and decision parameters based on their behavioral data.

### The present study

In this work, we present data from MDI and healthy comparison subjects (HCS) performing a binary-choice version of the multi-arm bandit task (Robbins, 1952). Each arm has a fixed and initially unknown reward rate (probability of reward per trial), though observers may have prior beliefs about the reward rate, and each observed outcome informs the decision-maker about the reward rate. To quantify the learning and decision-making processes in healthy human subjects and MDI, and to examine any subtle differences in the neural circuitry underlying these processes, we use a Bayesian modeling framework (DBM), in combination with five decision policies previously suggested in the literature (Win-Stay/Lose-Shift (WSLS), ε-Greedy, τ-Switch, Softmax, and Knowledge Gradient).

Given evidence of impaired learning in MDI (Miller and Cohen, 2001; Paulus et al., 2002), we hypothesized that relative to healthy comparison subjects (HCS), MDI would be more reliant on a simple learning-independent strategies (e.g., WSLS) and less reliant on more complex learning-dependent, principled strategies (e.g., Softmax). Moreover, given the reduced reward responsiveness of MDI to non-drug reinforcers (Goldstein and Volkow, 2011), we hypothesize that MDI subjects might have altered reward representation before and/or after observing reward outcomes on chosen options.

## Materials and methods

### Participants

The UCSD Human Research Protections Program and/or the Veterans Affairs San Diego Healthcare System (VASDHS) Internal Review Board approved the study protocol. All subjects gave written informed consent. Sixteen (40% female; mean age = 35.4) sober MDI were recruited from a 28-day inpatient Alcohol and Drug Treatment Program at the Veterans Affairs San Diego Healthcare System and Scripps Green Hospital (La Jolla, CA). To maintain sobriety during the program, participants were screened for the presence of drugs via urine toxicology. In addition, 16 healthy comparison subjects (HCS; 33% female; mean age = 37.1) were recruited via flyers, internet ads (e.g., Craigslist), and local university newspapers. HCS were selected to be matched in age and IQ with MDI. All subjects completed a clinical interview session behavioral session during which they completed the Bandit Task (these study procedures took place between the third and fourth week of treatment for MDI).

Lifetime DSM-IV Axis I diagnoses (including substance dependence) and Axis II antisocial personality disorder were assessed by experienced interviewers using the Semi Structured Assessment for the Genetics of Alcoholism (SSAGA) (Hesselbrock et al., 1999), a semi-structured interview that allows for quantification of lifetime drug use. Diagnoses were based on consensus meetings with a clinician specialized in substance use disorders (MPP) and trained study personnel. The following were exclusion criteria for all groups: (1) antisocial personality disorder; (2) current (past 6 months) Axis I panic disorder, social phobia, post-traumatic stress disorder, major depressive disorder; (3) lifetime bipolar disorder, schizophrenia, and obsessive compulsive disorder; (4) current severe medical disorders requiring inpatient treatment or frequent medical visits; (5) use of medications that affect the hemodynamic response within the past 30 days such as antihypertensives, insulin, and thyroid medication; (6) current positive urine toxicology test; and (7) history of head injuries with loss of consciousness for longer than 5 min. During evaluation, participants performed the North American Adult Reading Test (NAART; Uttl, 2002) as a measure of verbal intelligence (VIQ).

### Bandit task

**Figure 1:**
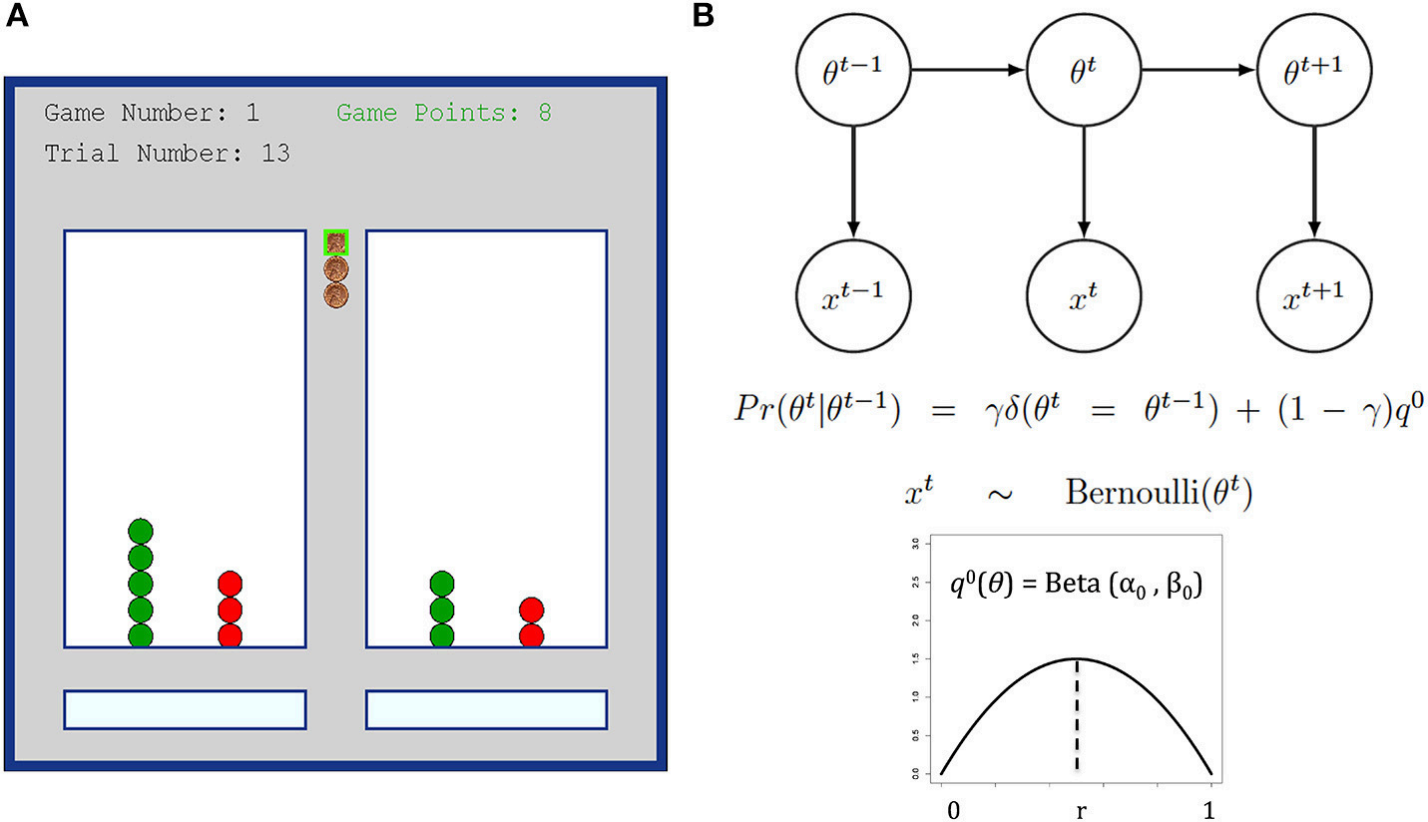
**(A)** Bandit task interface snapshot. Participants completed 20 games, each with 16 trials. **(B)** DBM illustration and the generative equations. The reward rate of each arm are assumed to be independently drawn at the start of a game from a Beta distribution *q*_0_ = Beta (α_0_, β_0_), fixed throughout the game, and with mean *r* = (α_0_)/(α_0_ + β_0_)_._ DBM assumes that subjects believe that the reward rate θ for any arm can reset on any trial with probability 1-γ, otherwise it is the same value as the last trial.

Participants completed 20 bandit games of 16 trials each on a computer. For each game, participants had 16 tokens (stacked in the middle of the screen) and had to assign one token on each trial to one of the two lottery arms. After placing each token, they either earned one point if the token turned green or zero points if the token turned red see Figure 1A. The reward rate for each arm was independent and identically sampled from a Beta distribution (α = β = 2) at the beginning of each game. In practice, the two arms always had different reward rates in each game, even though on average they had the same mean reward rate (0.5) and standard deviation (0.22). Participants were instructed at the beginning of the experiment that the rewards probability for the arms were independent, and were redrawn at the beginning of each game. They were further instructed to try to maximize the points earned over all trials and in all games. To additionally motivate the participants, we compensated subjects with a dollar amount proportional to their total points earned across all games at the end of the experiment (amounts paid ranged from 6 to $11).

### Modeling

We modeled trial-by-trial learning in humans using a form of the hidden Markov model, which we call the Dynamic Belief Model (DBM), which assumes the environmental statistics (i.e., reward rate for an arm in this task) to undergo unsignaled changes (Yu and Cohen, 2009; Zhang and Yu, 2013a,b). It includes the stationary case (the true experimental design) as a special case, whereby the probability of reward rate changing on each trial is exactly 0. We modeled the decision component using five competing decision policies: WSLS, ε-Greedy, τ-Switch, Softmax, and Knowledge Gradient. In the following, we first describe the statistical learning model, then the decision policies, and finally the model-selection procedure for identifying individual decision strategies and group learning parameters.

#### Dynamic belief model

As a simple variant of a hidden Markov model, the generative model assumes that on each game, the two arms have reward rates, θ_m_, *m* = 1 or 2, each independently generated from the generic Beta prior distribution q^0^(θ_m_) = Beta (α_0_, β_0_) with mean *r* = (α_0_)/(α_0_ + β_0_). For simplicity, we assumed that the sum of its two parameters (which controls variance) to be fixed at α_0_ + β_0_ = 4. We also assumed that the reward rate for each arm has a probability γ of staying the same as last trial, and a probability (1-γ) of being independently reset and re-drawn from q^0^ on any trial, hence embodying the assumption of non-stationarity in the Dynamic Belief Model see Figure 1B. We call γ the stability parameter, since larger γ results in a more stable arm that changes reward rates less frequently (γ = 1 is a special-case arm that never changes reward rate at all).

Given the above generative model, we can use standard Bayesian probability theory to compute the posterior distribution over reward rates of the two arms on each trial, after making an observation. We use the notation *q*${}_{m}^{t}$ (θ${}_{m}^{t}$) := Pr(θ${}_{m}^{t}$|x^*t*^) to denote the posterior probability distribution over the reward rate for the *m*th arm on the *t*th trial, denoted θ${}_{m}^{t}$, given the observed sequence of successes and failures from all previous trials, denoted x^*t*^: = (x^1^…x^*t*^). On each trial *t*, the observer's iterative prior distribution marginalizes over uncertainty about whether there has been a reward rate change on the current trial, and is therefore a mixture of last trial's posterior and the generic prior:

$$\mathrm{Pr}(\theta_{m}^{t}=\theta |{\text{x}}^{t-1})=\gamma q_{m}^{t-1}(\theta )+(1-\gamma )q^{0}(\theta )$$

To update the posterior after the current trial, for the chosen arm only (assuming it is the *m*th arm), having observed the outcome *R*${}_{m}^{t}$ (1 for a reward, 0 for no reward), the new posterior distribution for the chosen arm can be computed via Bayes' rule:

$$q_{m}^{t}(\theta_{m}^{t})\sim \mathrm{Pr}(R_{m}^{t}|\theta_{m}^{t})\mathrm{Pr}(\theta_{m}^{t}|{\text{x}}^{t-1})$$

whereas, the posterior for the un-chosen arm is the same as the prior at the beginning of the current trial (since there has been no new observation). The mean of the prior distribution, $\mu_{m}^{t}$, is what we call *estimated reward rate* for arm *m*.

In the actual experimental design, the reward rates were fixed. This is one possible, special case setting also captured by the DBM, by assuming the probability of the reward rate changing on any trial is 0 (γ = 1), which we call the Fixed Belief Model (Yu and Cohen, 2009; Zhang and Yu, 2013b).

#### Decision policies

In the cognitive science and reinforcement learning literatures, a number of decision policies with varying levels of complexity have been used to model human bandit choice (Daw et al., 2006; Steyvers et al., 2009; Zhang et al., 2014). These policies can be conceptualized as underlying the choice of a goal-directed action based on the individual's current beliefs and knowledge of their environment. Here, we considered five models, ordered below by increasing complexity: Win-stay/Lose-shift (WSLS), τ–Switch, ε-Greedy, Softmax, and Knowledge Gradient. WSLS is a simple, learning-independent heuristic policy, which stays with the last chosen arm after a reward with probability γ^w^ and switches to the other arm after a loss (no reward) with probability γ^l^ (Robbins, 1952). τ*-Switch* is another learning-independent policy that assumes that the decision-maker uses a fast-and-frugal heuristic for their choice selection depending on the counts of previous successes and failures for both arms. They choose randomly when the two arms have equal counts of previous successes and failures, namely S_1_ = S_2_ and F_1_ = F_2_. The second situation is when one arm is better/worse than the other; for example, Arm 1 is better (or Arm 2 is worse) if S_1_ > S_2_ while F_1_ ≤ F_2_, or F_1_ < F_2_ while S_1_≥S_2_, and the model chooses the better arm with (a large) probability γ^τ^. When one arm has both more previous successes and failures than the other, it is an “exploit” option, and the other arm is an “explore” option; in this situation, the model chooses the explore option with probability γ^τ^ if the current trial is before the switch point τ, otherwise chooses the exploit option with probability γ^τ^ if the current trial is after τ. A detailed description of this model can be found in Lee et al. (2011). γ^τ^ is a parameter of “accuracy of execution,” which captures the proportion of choices that are consistent with either the exploration or exploitation policy on any given trial (Steyvers et al., 2009). ε-Greedy assumes that one chooses the alternative with the greatest estimated reward rate with probability 1-ε on each trial, but chooses randomly among the remaining arms with probability ε (Barto, 1998). The Softmax decision policy assumes that the decision-maker chooses among the options with probabilities related to the inferred reward rates of the respective arms, but often with exaggerated ratios (over-matching) compared to the estimated reward rates, but typically not linearly (Luce, 1959). Here, we assumed that the choice probabilities are normalized polynomial functions of the estimated reward rates, with polynomial parameter *b*, e.g., Pr(choosing arm 1) = μ${}_{1}^{\text{b}}$/(μ${}_{1}^{\text{b}}$ + μ${}_{2}^{\text{b}}$), so that when *b* approaches infinity, the maximally rewarding option is always chosen (maximizing), when *b* is 1, it is probability matching, and when *b* is 0, the arms are chosen randomly (with equal probability. Knowledge Gradient or KG (Frazier et al., 2008; Ryzhov et al., 2012; Zhang and Yu, 2013b) is the most sophisticated among the heuristic policies we consider here. In its original formulation, KG is a deterministic policy that chooses the arm with the highest combined gain of immediate reward (first term in the equation below) and longer-term “knowledge gain” (second term), with the linear tradeoff parameterized by the distance to horizon (e.g., fewer trials left results in less emphasis on “knowledge gain”). Namely, the decision rule is

$$D^{KG,t}=argmax_{m}\mu_{m}^{t}+(T-t-1){\text{v}}_{m}^{KG,t}$$

where *v*${}_{m}^{KG,t}$ = E[*max* θ^*t*+1^|*D*^*t*^ = *m, q*^*t*^]-*max* θ^*t*^ is the approximate value function for choosing arm *m* on trial *t*, under the current belief state *q*^*t*^. This formulation is similar to the optimal policy except that the second term in *KG* approximates the value of exploration (second time) by using a lower-bound, that attained by allowing only one more exploratory step and exploitation thereafter, whereas the real optimal policy would also consider the possibility of further exploratory choices, which involves a much more expensive computation. Note that *KG* does not actually choose to exploit after one more exploratory step, it merely estimates the *value* of further exploration using this computational assumption. It is interesting to note that *KG* is equivalent to the optimal policy of explicitly maximizing cumulative gain, when the reward rates are assumed to be fixed (γ = 1) and there are only two arms (Frazier et al., 2008), however, they are not equivalent in the problem here under the DBM (non-stationary) assumptions. In this work, we extended the original formulation of *KG* by adding a free parameter γ^kg^ that turns this deterministic policy into a probabilistic policy, such that Pr(choosing arm *m*|*D*^KG, t^ = *m*) = γ^kg^, so as to match the other algorithms better in terms of the number of free parameters. Both *KG* and the optimal policy increasingly favor exploitation over exploration with fewer trials left; as we did not see this tendency in subjects' behavior in both a previous study (Zhang and Yu, 2013b), and the current data set (results not shown), we do not explicitly consider the optimal policy here. Detailed description of the optimal policy that KG approximates under stationary bandit setting can be found in Zhang and Yu (2013b).

### Bayesian model comparison

As a compromise between model specificity and statistical power, we assumed individuals in the same group (i.e., MDI or HCS) to share the same DBM stability parameter γ and the mean of the generic prior *r.* However, we assumed that individuals may differ in their decision policy, both in terms of which policy and what parameter setting. Specifically, for each fixed pair of DBM parameter values (γ, *r*), where γ and *r* each varies from 0 to 1 in increments of 0.1 (they are bounded by 0 and 1), we can compute for every subject a sequence of trial-wise prior distributions over reward rates for the two arms, based on his/her actual sequence of choices and observations (reward or no reward), and thus a likelihood of observing the subject's choice on each trial for each policy (and each parameter setting of each policy). This would allow us to compute a joint likelihood of all data (all choices of all subjects) by multiplying the likelihood of each observation (a subject's choice on one trial), and thus a means for comparing estimates of (γ, *r*) between MDI and HCS, and the decision policies and policy parameters across subjects.

**Figure 2:**
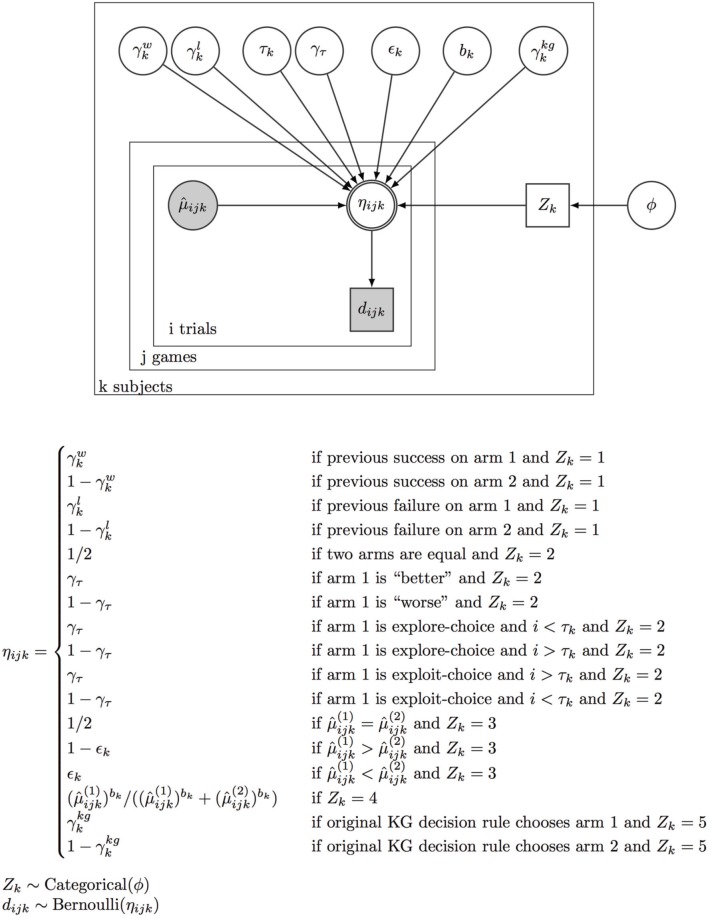
**Bayesian model comparison**. We simultaneously infer the latent model usage and model parameters for all five different strategic decision-making models, including two learning-independent heuristic models (WSLS, τ-Switch), two learning-dependent heuristic models (ε-Greedy, Softmax), and Knowledge Gradient, based on observed data (subjects' actual choices and outcomes). See Materials and Method for more details. In the Bayesian graphical model, a node is double-bordered if it is deterministic (on its parents), otherwise stochastic; a node is gray if it is known/observed, otherwise unknown and to be inferred. Circle node is continuous, whereas rectangular node is discrete. η is the probability for choosing arm 1 for subject k, at trial i of game j. This choice probability is deterministically dependent on the estimated reward rates and the decision policy.

However, it is very computation- and memory-intensive to compute a joint likelihood for all model parameters (e.g., by discretizing each parameter) for all data. We therefore specified a Bayesian graphical model of how the data (subjects' observed choices) are generated from the underlying model parameters see Figure 2, and use WinBUGS (Spiegelhalter et al., 2003), to sample (using Markov chain Monte Carlo or MCMC) from the full joint posterior distribution over all the decision-policy parameters conditioned on the observed data. Specifically, we assumed each subject *k* utilizes policy *l*, *z*_*k*_ = *l*, for all trials with a categorical probability distribution that has a Dirichlet prior distribution, Dir(1,1,1,1,1), which yields a marginal prior probability of 1/5 for each policy, and each decision policy parameter has a prior distribution that is uniform over the unit interval (γ^w^, γ^l^, ε, and γ^τ^) except for *b*, the Softmax parameter, which has a very flat Gamma prior with support over R^+^ (mean = 10, std = 10). Because all the priors are flat (or nearly flat), the posterior surface is approximately proportional to the data likelihood, but represented by samples, such that the likelihood (posterior probability) for a region of the parameter space is reflected in the relative number of samples. We compared the “average” likelihood of different settings of (γ, *r*) for each group (by marginalizing over the uncertainty associated with the choice of decision policies and their parameters), or the “average” likelihood of each policy for every subject (by marginalizing over the uncertainty associated with the parameters of each policy). This constitutes a form of Bayesian model comparison, which has the convenient feature of automatic penalization of model complexity.

Based on the specified generative model, for each setting of (γ, *r*), we obtained from WinBUGS 2000 samples (two MCMC chains, each containing 1000 samples with a burn-in period of 1000 samples, and using standard checks for convergence, Gelman and Rubin, 1992) of model parameters, each sample containing the setting of the indicator variable *z*_*k*_ specifying subject *k*'s decision policy, and the parameter setting of the relevant policy; WinBUGS also returns the data likelihood associated with each sample. To identify the DBM parameters (γ, *r*) for each group (MDI or HCS), we computed the marginal data likelihood by integrating out uncertainty over *z*_*k*_ and all decision policy parameters [i.e., adding up the likelihood of all samples for (γ, *r*)] and use the maximal marginal likelihood estimates for (γ, *r*). Having fixed (γ, *r*) for each group (MDI or HCS), we could then identify the policy used by each subject, ẑ_*k*_, by again finding the maximal marginal likelihood estimates for *z*_*k*_ (i.e., adding up the number of samples for each setting of the categorical variable *z*_*k*_). Finally, to identify the decision policy parameter used by subject *k*, we considered all samples where *z*_*k*_ = ẑ_*k*_, and found the policy parameter setting (sample) with the highest likelihood.

### Group comparison statistics

**Figure 3:**
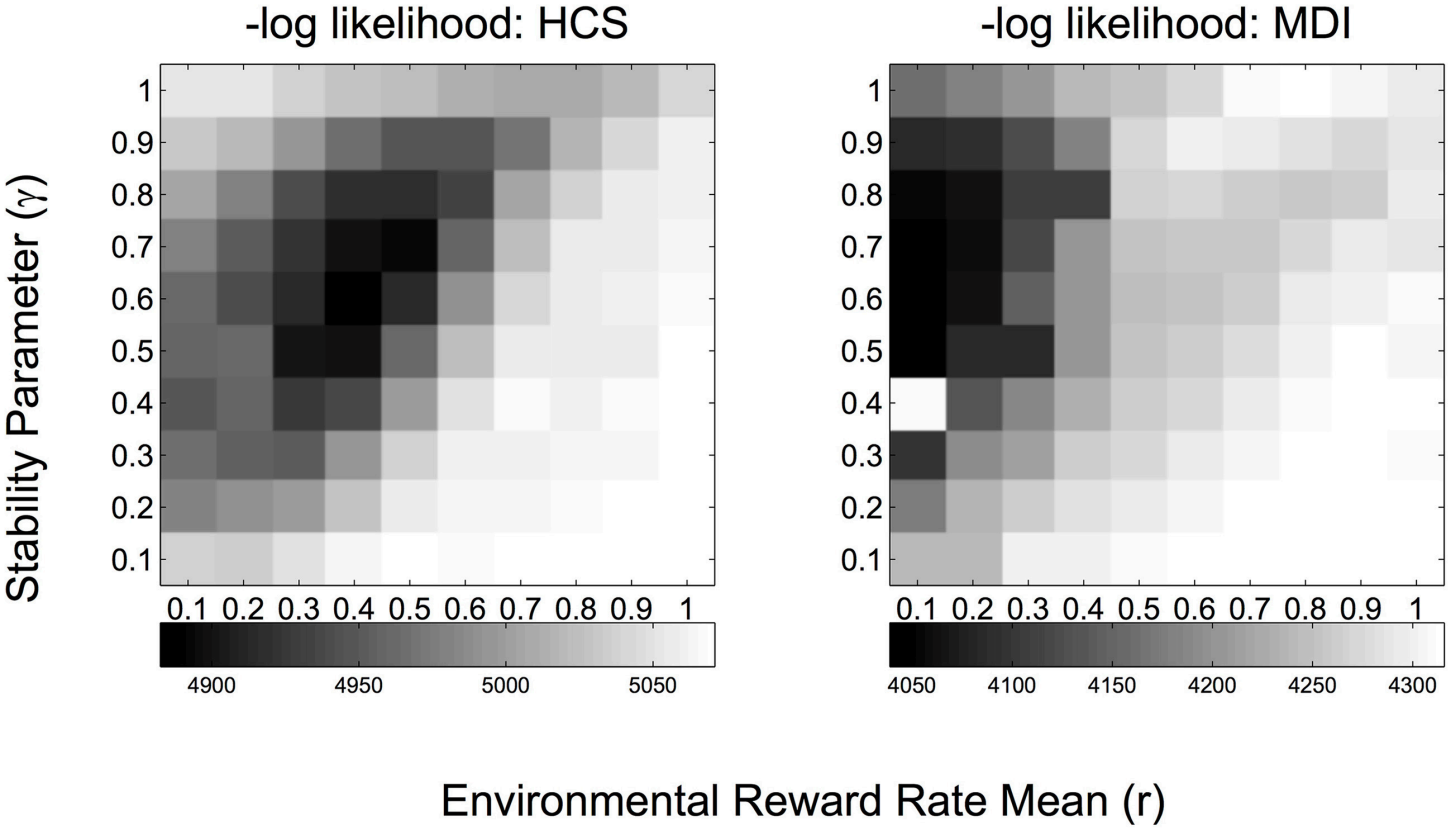
**-Log likelihood grayscale plots for each pair of DBM parameter values (γ, *r*) fitted at the group level (MDI, methamphetamine dependent individuals; HCS, healthy comparison subjects)**. Values represent −2^*^log likelihood, where the likelihood is marginalized over uncertainty associated with the decision policy utilized by each subject and the parameter setting of the policy. Darker color indicates lower log likelihood, thus better fit.

For behavioral variables with repeated measures (e.g., trial-wise reaction times and game points), we fit hierarchical generalized mixed-effect linear models treating subject as a random factor (with varying intercepts in one model, and also varying slopes in another model) and other variables as fixed effects (Baayen et al., 2008). For group comparison of individually fit parameters, independent two-sample *t*-tests were used. To compare the learning (DBM) parameters (γ, *r*) between the two groups, we estimated the “mean” and “variance” of γ, *r*, by resampling from the marginal data likelihood (shown as grayscale maps in Figure 3), or equivalently the marginal posterior distribution assuming a uniform prior over (γ, *r*), using one MCMC chain of length 10^7^. The mean was estimated by the sample mean, the variance was estimated by the sample variance. These estimates were then used, together with the actual group sizes (16) for MDI and HCS, to construct the 95% CI (t-critical value of 2.04 for a two samples *t*-test; df = 16+16-2 = 30).

### MRI image acquisition and voxel-based morphometry (VBM)

High-resolution *in vivo* structural MR-images (T_1_-weighted spoiled gradient recalled [SPGR] imaging, TR = 8 ms, TE = 3 ms, slices = 172, FOV = 25 cm, approximately 1 mm^3^ voxels) were acquired in all subjects on a 3.0 Tesla Signa EXCITE scanner (GE Healthcare, Milwaukee, WI). Optimized voxel-based morphometry (VBM) was performed with the FSL-VBM pipeline (Douaud et al., 2007); http://fsl.fmrib.ox.ac.uk/fsl/fslwiki/FSLVBM) using FSL tools (FSL-4.1.6; (Smith et al., 2004). Optimized VBM uses an iterative approach to segmentation and normalization that results in a more accurate identification of gray and white matter (Good et al., 2001). Brains were first automatically extracted from the skull with BET (Smith, 2002). Tissue-types (i.e., gray matter, white matter, CSF) were then segmented with FAST4 (Zhang et al., 2001). The gray matter images were aligned with the MNI-152 standard space by affine registration (d.f. = 12) using the FLIRT tool (Jenkinson et al., 2002) followed by non-linear registration using FNIRT (Andersson et al., 2007) and averaged to create a study-specific template. Segmented gray matter images in native space were then re-registered to this template. To preserve information about absolute volume, partial volume images were modulated by the non-linear component of the Jacobian determinants generated during spatial normalization thus obviating the need to correct for total intracranial volume (Scorzin et al., 2008). To make the residuals in subsequent analyses conform more closely to a Gaussian distribution and to account for individual differences in brain anatomy, the modulated GM images were smoothed with an isotropic Gaussian kernel, σ = 3 mm ≈ 7.06 mm FWHM. The average modulated gray matter volume was extracted from 70 cortical and subcortical regions of interest (ROIs). The ROIs were defined by a maximum probability map based on the Talairach atlas. The construction of these ROIs are described elsewhere (Fonzo et al., 2013; Ball et al., 2014).

## Results

### Behavioral measures

Figure 1A illustrates the bandit task we used in this study. Combining all subjects, we found a negative linear relationship between reaction times (RT) and game number (*B* = −12 ms, *t* = −3.6, *p* < 0.001, model omnibus test: χ^2^ = 35.9, *p* < 0.001; Mean RT = 1428 ms), such that individuals made faster decisions as they had more experience with the bandit task. However, neither the group main effect (χ^2^ = 0.46, *p* = 0.50) nor the group × game interaction (χ^2^ = 0.86, *p* = 0.35) were significant, i.e., MDI did not differ from HCS on their general latency to select an option nor on their decrease in latency for later trials. In general, we did not find earnings to vary significantly as a function of game number (χ^2^ = 0.02, *p* = 0.89; Mean Game Points = 8.9)—in particular, subjects did not improve in their performance as they had more experience. Correspondingly, we also found no group difference in total earnings, both in terms of overall group effect (χ^2^ = 0.01, *p* = 0.93) or group × game interaction (χ^2^ = 0.03, *p* = 0.86). Thus, MDI and HCS had similar overall performance in the bandit task.

### Learning model

The best data-fitting parameters for the learning model (DBM, see Figure 1B) were inferred for each group: these consist of estimates for the prior expectation of reward rate, $\hat{r}=$ (â)/($â+\hat{b}$*)*, and the stability parameter, $\hat{\gamma }$, which also controls the effective exponential memory window size and thus can be thought of as a discount rate parameter (larger γ = less assumed volatility in reward rates = longer memory window = slower/less discounting, see Yu and Cohen, 2009). Figure 3 shows the logarithm of the marginal likelihood values for each setting of (γ, *r*) where each variable varies between 0 and 1 in increments of 0.1, i.e., how well different settings of (γ, *r*) can account for all the observed choices of all subjects in a group (MDI or HCS), after marginalizing over uncertainty about the hidden parameters that specify which decision policy each subject uses and the parameter setting of that policy (see Materials and Methods for details). As shown in Figure 3, we found that MDI and HCS have similar estimated stability parameter $\hat{\gamma }$ (MDI: Mean = 0.60; HCS: Mean = 0.59; CI95%: −0.005 < μ_HCS_–μ_MDI_ < 0.006). Both HSC and MDI behave as though they believe the environment to be changeable—in fact, at approximately once every 1/(1-$\hat{\gamma }$) = 1/(1-0.6) = 2.5 trials—instead of assuming the reward rates to be static, which was the “true” experimental design. The estimated ∩γ is smaller than values found in most other tasks, which tends to be around 0.7–0.8 (Yu and Cohen, 2009; Ide et al., 2013; Yu and Huang, 2014; Zhang et al., 2014; Ma and Yu, 2015), and may potentially be due to the longer inter-trial interval used in this task (temporal discounting may be influenced also by absolute time, not only discrete trials as assumed in DBM).

Unlike the estimates for stability/volatility, we found that MDI and HCS *do differ* in their prior beliefs about mean reward rate (MDI: Mean = 0.11; HCS: Mean = 0.40; CI95%: 0.28 < μ_HCS_–μ_MDI_ < 0.31), such that MDI seem to have lower prior belief of receiving reward from any lottery arm (probability = 0.11) relative to HCS (probability = 0.40), i.e., MDI individuals are overall more pessimistic about the same reward environment than HCS.

### Decision policy

**Figure 4:**
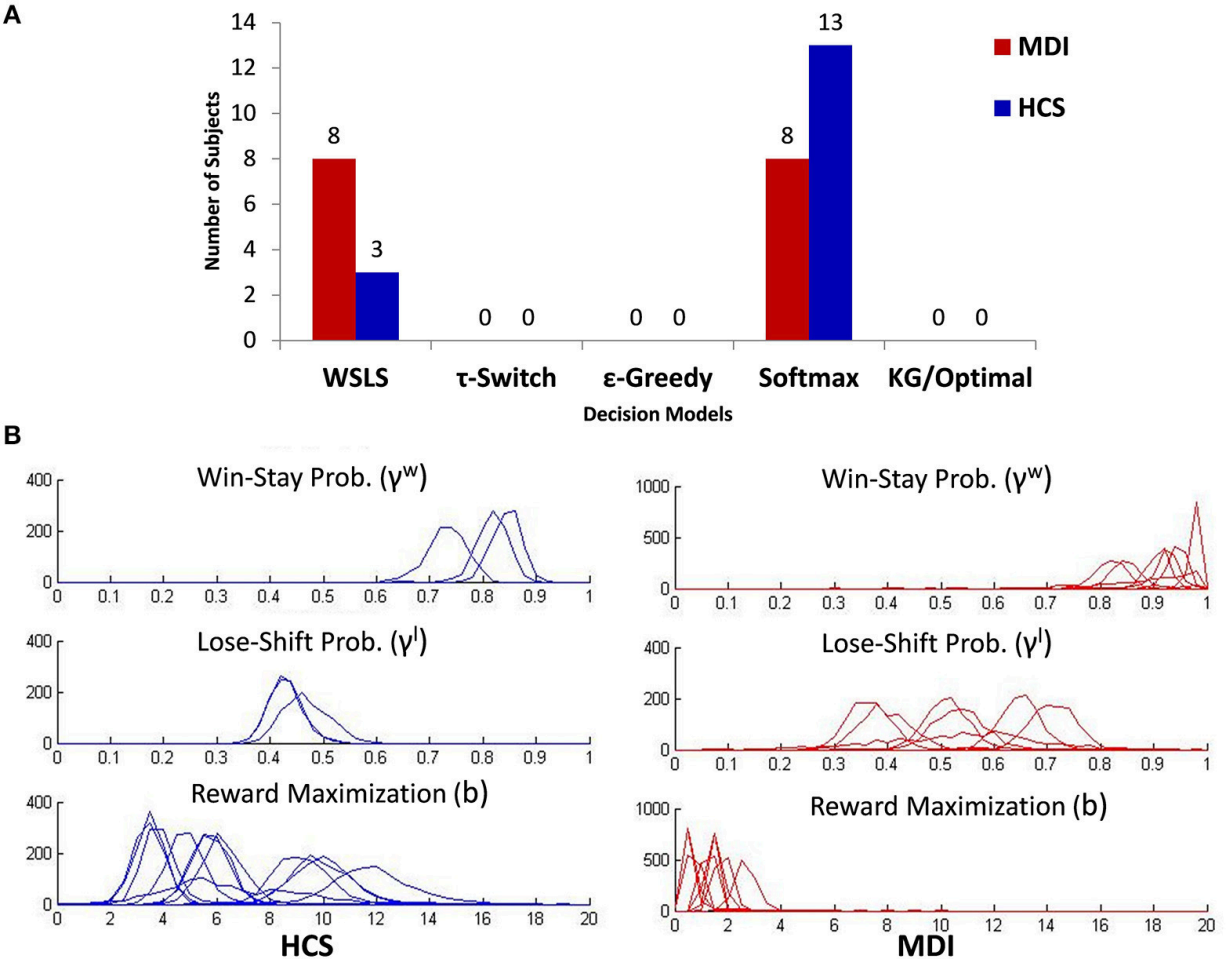
**(A)** Classification of all subjects by group (MDI, methamphetamine dependent individuals; HCS, healthy comparison subjects) and model usage based on posterior mode of the *z*_*ijk*_ parameter. **(B)** Posterior distributions for the parameters associated with the strategic control model used by participants: Win-Stay/Lose-Shift (WSLS; γ^w^ γ^l^: top two graphs) and Softmax (*b* parameter; bottom graph). The y-axis depicts a histogram of the counts of MCMC samples obtained for each of these parameters for each individual (overlaid) identified to be most likely to be using the corresponding policy.

To identify the decision policy utilized by each individual, and to estimate the relevant model parameter(s), we first fixed the DBM parameters at the values estimated for each group (see previous section), found the policy for each individual that yield the highest marginal likelihood for the observed choice data for that subject (marginalized over parameter settings), and then estimated the policy parameter setting that achieves the highest likelihood. We found that WSLS or Softmax to best explain each participant's data, or in other words, Knowledge Gradient, τ-switch, and ε-greedy are not as good at predicting any participant's behavioral data. While WSLS and Softmax were each found to be the best fitting policy for some individuals in each group, there is was a statistical trend toward a higher proportion of MDI relying on the learning-independent WSLS (8/16 = 50%) compared to HCS (only 3/16 = 19%; χ^2^ = 3.46, *p* = 0.06). Conversely, while a majority of HCS used a Softmax strategy (81%), only half of MDI used such model (50%; see Figure 4A).

Based on this result, we only provide group comparisons of the parameter values of the two best-fitting models (γ^w^ and γ^l^ for WSLS; and b for Softmax). Figure 4B shows the posterior distribution over model parameters (assuming uniform priors) for different subjects (overlaid), where each figure only contains the posterior of individuals whose choices are best explained by the corresponding policy. In practice, the posterior distributions are approximated by the counts of MCMC samples obtained by WinBUGS for each of these parameters for each individual (see Materials and Methods). In the following, we used only maximum-likelihood estimates (MLE) for decision policy parameters. Among individuals using WSLS, MDI, and HCS did not differ in their tendency to lose-shift, i.e., switching arms after not receiving a reward (MDI: Mean γ^l^ = 0.54; HCS: Mean γ^l^ = 0.45, *t*_(9)_ = 1.2, *p* = 0.25), but MDI had significantly higher tendency than HCS to win-stay, i.e., choosing the same arm after receiving a reward (MDI: Mean γ^w^ = 0.91; HCS: Mean γ^w^ = 0.81, *t*_(9)_ = 2.7, *p* = 0.03). Among those using the Softmax strategy, relative to HCS, MDI had a significantly lower reward maximization parameter (MDI: Mean *b* = 1.58; HCS: Mean *b* = 6.99, *t*_(19)_ = 5.6, *p* < 0.001), indicating that MDI select actions more like *matching*, while HCS act more like *maximizing*, which has also been found for healthy individuals in other learning and decision-making task (Zhang et al., 2014).

### Relationship between computational parameters and gray matter brain volumes

**Figure 5:**
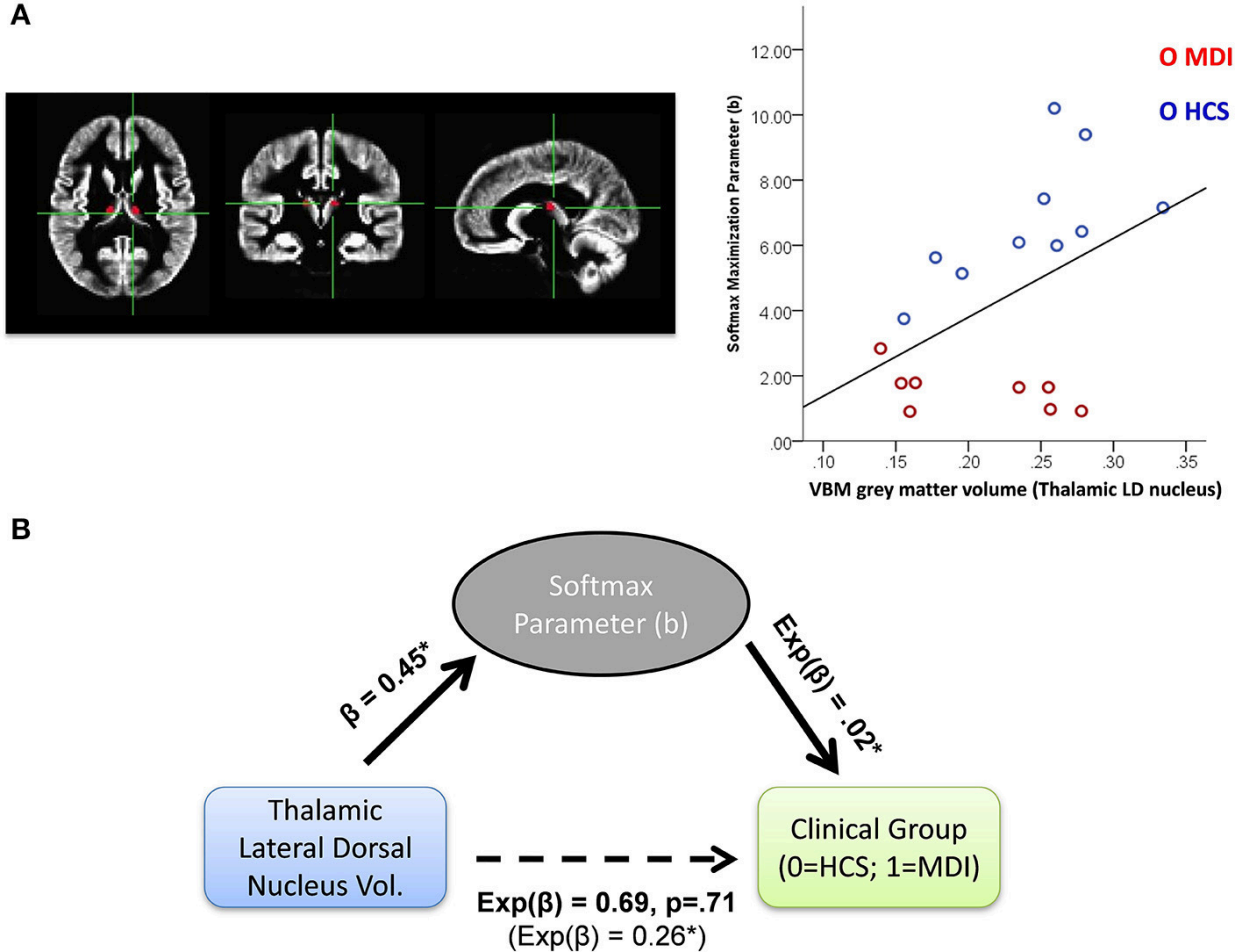
**(A)** Left panel: thalamic lateral dorsal nucleus (LD) ROI in axial, coronal, and sagittal planes; Right: correlation between Softmax b paramter and VBM based gray matter volume in the thalamic LD in Softmax users (*N* = 17). **(B)** Mediation analysis with hierarchical generalized linear models (with a logit link function for models predicting clinical group). Softmax *b* parameter was found to fully mediate the negative relationship between thalamic LD gray matter volume and likelihood to be MDI vs. HCS (MDI, methamphetamine dependent individuals; HCS, healthy comparison subjects); ^*^p < 0.05.

To further investigate the potential neural substrate of individual behavioral differences, we conducted exploratory correlational analyses within each set of model users (i.e., WSLS and Softmax users) between individual parameter values and average VBM gray matter relative volumes for 70 ROIs. No association was found between WSLS parameters (γ^w^ and γ^l^) and anatomical gray matter volumes. Within Softmax users, we found a positive association between the reward maximization b parameter and gray matter volumes of the thalamic lateral dorsal nucleus (LD; *r* = 0.45, *p* < 0.05; see Figure 5A).

Given the strong relationship between clinical group status and Softmax *b* parameter, we further investigated the relationship between clinical status and gray matter volume in this thalamic region and the potential mediating role of the Softmax parameter in this relationship. To do so, we used a hierarchical regression method, with both linear and logistic regressions to accommodate for the dichotomous clinical group variable (Baron and Kenny, 1986). As expected, a first model showed that higher Softmax *b* parameter was associated with a lower likelihood to belong in the MDI group (ominibus χ${}_{\left(1\right)}^{2}=17.1$, *p* < 0.001; odd ratio = 0.02). Another model showed that higher thalamic LD gray matter volume was associated with a lower likelihood to belong in the MDI group (ominibus χ${}_{\left(1\right)}^{2}=4.9$, *p* < 0.05; odd ratio = 0.26). Thalamic LD gray matter volume was positively related to Softmax b parameter, *F*_(1, 18)_ = 4.2, *p* = 0.05 (beta = 0.45). Importantly, adding Softmax b parameter as a second predictor of clinical status removed the effect of thalamic LD gray matter volume (*p* = 0.71), leaving the Softmax parameter as the only significant predictor of clinical status (ominibus χ${}_{\left(1\right)}^{2}=17.2$, *p* < 0.001; odd ratio = 0.03), consistent with a full meditation of the Softmax parameter (see Figure 5B).

## Discussion

In this study, we applied a probabilistic learning and decision-making model human choice behavior data in a bandit task, in order to investigate cognitive differences in learning and decision-making between recently sober MDI and HCS. To model the representation and updating of individuals' beliefs, we used the Dynamic Belief Model, a Bayesian iterative inference model which assumes the environment to undergo unpredictable and discrete changes (Yu and Cohen, 2009). The decision-making component was modeled with a set of five well-established decision policies from the cognitive science and reinforcement learning literatures, including Win-stay/Lose-shift (WSLS), ε-Greedy, τ-Switch, Knowledge Gradient, and Softmax. To our knowledge, this is the first study using such hierarchical Bayesian approach to assess reward processing in a clinical population such as MDI.

### Bandit choice behavior

The bandit task has been a popular behavioral paradigm for studying the exploration-exploitation tradeoff, as the task involves a potential conflict between actions that maximize the short-term potential of immediate reward and the long-term gain of information that maximizes total rewards. Our modeling framework decomposes the task into a learning component, which consists of learning about initially unknown reward rates for the two arms in each game, and a decision component, which consists of choosing an arm on each trial based on previous observations and any prior beliefs. We found that the learning algorithm that best describes each of HCS and MDI subjects (in the latter group, only those who show learning) indicates the subjects to be assuming the reward rate statistics to be changing on a relatively fast timescale (about once every 2.5 trials), despite the experimental reward rates to be actually constant in a game, but consistent with healthy human choice behavior in a variety of behavioral tasks. A consequence of this peculiar non-stationarity belief is that a subject's belief (reflected in his/her choice) on the current trial is strongly influenced by the outcome of the most recent trials in the past, and exponentially less by outcomes farther into the past (Yu and Cohen, 2009), producing what is classically known in psychology as *sequential effects*. In terms of the decision policy, we found that every HCS and MDI subject was best described as utilizing a heuristic Softmax policy or the even the simpler, learning-independent WSLS policy. Unlike the more sophisticated Knowledge Gradient policy (and of course the optimal policy, see Zhang and Yu, 2013b), Softmax does not explicitly assess the relative value of future exploratory gain vs. immediate exploitative gain, but rather uses a single fixed parameter (*b* in this paper) to heuristically “loosen” up the choice policy relative to the estimated reward rates of the given options. Consequently, one important difference between Softmax and Knowledge Gradient (and also the optimal policy) is that the former policy is insensitive with respect to the number of trials left (known as the horizon in reinforcement learning literature), while Knowledge Gradient (and the optimal policy) weigh exploratory gain less relative to exploitative gain as the horizon gets closer and there is less time left to take advantage of any additional information gained. This insensitivity to horizon finding is consistent with what we previously found for healthy human subjects in the bandit task (Zhang and Yu, 2013b). However, in addition to these coarse similarities between MDI and HCS in both learning and decision-making, there are also some subtle but important differences, as we detail below.

### Learning alterations in MDI

Relatively fewer MDI (50%) than controls (81%) used the Softmax decision policy, instead favoring the WSLS policy. Thus, less MDI were likely to use a learning-supported strategy, which uses estimated reward rates for all arms, and instead used a myopic heuristic relying solely on previous trial outcome. This result is consistent with research suggesting that MDI are impaired in learning and updating their knowledge of the environment and generally have difficulties “seeing the big picture.” For instance, along with weaker recruitment of neural regions associated with learning such as the DLPFC and anterior insula, impairments in working memory (Chang et al., 2002) and sequential decision-making (Rogers et al., 1999; Paulus et al., 2002) have been noted in this population, along with difficulties detecting trends and integrating new information to predict future outcomes (Aron and Paulus, 2007). The present results suggest such deficits may also manifest in the type of decision policies implicitly chosen to make reward-based decisions.

### Non-drug reward hyposensitivity in MDI

We also found that among individuals using a Softmax strategy, MDI had on average lower reward maximization (*b*) parameter values compared to HCS. Because this parameter reflects the weight given in the action policy to the options with highest predicted reward rates, this individual metric may be conceptualized as an individual's reward sensitivity bias, independently of their expectations of reward. Interestingly, such parameter value tended toward a value of 1 in MDI, which is equivalent to the special case of probability matching for this policy (i.e., choosing arms in the same proportion as the estimated reward rates). Thus, those MDI who are presumably good learners (i.e., using a DBM learning-based strategy) showed lower preference toward options they estimated to have the highest pay-off rate. Further consistent with this reward hyposensitivity, MDI as a group appears to have a lower prior expectation of reward in their environment (10% reward rate) relative to controls (40%).

These findings are congruent with evidence of decreased incentive salience and altered reward sensitivity toward non-drug rewards observed in substance users (Chang et al., 2002). For instance, relative to control subjects, cocaine-addicted individuals showed reduced activation of the left OFC for high gains in a forced-choice task under three performance-based monetary reward conditions (Goldstein and Volkow, 2011). In this study, cocaine abusers were also less sensitive to differences between monetary rewards in left OFC and in DLPFC, with a majority exhibiting flat value ratings of all monetary amounts received in the task. Similarly, a recent study found that MDI had weaker neural responses to the anticipation of a pleasant interoceptive stimulus with mechano-receptive C-fiber stimulation (i.e., forearm and palm pleasant touch), which was apparent in the anterior insula, dorsal striatum, and thalamus (Goldstein et al., 2007). Together with the present findings, this research points to a decreased sensitivity to non-drug rewards in MDI, which has been linked to negative emotionality and thus may pause a challenge for the therapeutic rehabilitation of these patients (Koob and Le Moal, 2001; Goldstein and Volkow, 2011; May et al., 2013). Thus, future studies should examine how stimulant and other substance-dependent individuals respond to non-drug related reinforcers. A computational approach, as shown here, may be particularly useful to tease apart subtle reward sensitivity and strategic alterations based on behavioral data, without the use of cost-heavy methods such as fMRI.

Finally, using VBM analysis of structural brain scans, we found a positive correlation between participant's Softmax reward maximization parameter and gray matter volume of the thalamic LD nucleus. Importantly, MDI also had lower LD gray matter volumes relative to HCS, and this relationship was mediated by the Softmax reward maximization parameter. The lateral dorsal nucleus is part of the limbic system and has been implicated in emotional processing. It receives input from the hippocampal gyrus (Leventhal et al., 2010) and is connected reciprocally with the cingulate gyrus, a region involved in decision-making and the processing of conflict and expectancy violation (Somerville et al., 2006; Alberstone, 2009). Moreover, through its connections to the retrosplenial area as well as the pre− and parasubiculum, the LD is thought to be involved in the integration of directional information for spatial navigation (Kennerley et al., 2011) and may contribute to supporting episodic memory and mental imagery of future events (Kaitz and Robertson, 1981; Maguire et al., 2003). Thus, while these data are preliminary and require confirmation in larger samples, our findings suggest that this thalamic structure could also play an important role in modulating reward sensitivity and choice behavior during sequential goal-directed decision-making.

## Summary

Using a computational approach, we found evidence of cognitive abnormalities underlying reward-based learning and decision-making in MDI. Such alterations were apparent at several levels, including beliefs about hidden reward rates in the environment (MDI had lower prior expectations of reward), the type of strategy/decision policy used (more MDI relied on a myopic learning-independent strategy), and the extent of bias toward choosing the options believed to have the highest reward rates (MDI exhibited lower reward maximization bias based on the Softmax policy).

Given the absence of group differences on coarse behavioral measures, such as reaction times and points earned in the game, our results suggests that a sophisticated computational modeling approach can be a powerful neuropsychological tool to capture a combination of subtle learning and strategic abnormalities in clinical populations. Therefore, such models could be particularly useful to more precisely and comprehensively identify behavioral and neurological markers of cognitive deficits in substance-using individuals, which in turn may help develop better clinical risk prediction models. It will be critical in future research to identify how these computationally derived cognitive biases can predict the development and maintenance of the addiction cycle, including the recurrence of cravings and drug seeking behavior. For instance, while all cognitive abnormalities identified here are likely to partly contribute to behavioral dysfunction in MDI, a decreased responsiveness to non-drug rewarding stimuli may play a prominent role in perpetuating anhedonia and negative emotionality, which in turn may lead to craving and increased likelihood of relapse. Behavioral interventions aimed at boosting healthy reward responsiveness and positive affect are thus worth investigating as possible tools for promoting substance abuse prevention and recovery.

## Funding

This study was funded by NIDA grant F32DA036959 (to KH).

### Conflict of interest statement

The authors declare that the research was conducted in the absence of any commercial or financial relationships that could be construed as a potential conflict of interest.
